# Comparative transcriptomics of albino and warningly‐coloured caterpillars

**DOI:** 10.1002/ece3.7581

**Published:** 2021-05-02

**Authors:** Juan A. Galarza

**Affiliations:** ^1^ Dpartment of Biological and Environmental Science University of Jyväskylä Jyväskylä Finland

**Keywords:** aposematism, *Arctia plantaginis*, gene expression, melanin

## Abstract

Coloration is perhaps one of the most prominent adaptations for survival and reproduction of many taxa. Coloration is of particular importance for aposematic species, which rely on their coloring and patterning acting as a warning signal to deter predators. Most research has focused on the evolution of warning coloration by natural selection. However, little information is available for color mutants of aposematic species, particularly at the genomic level. Here, I compare the transcriptomes of albino mutant caterpillars of the aposematic wood tiger moth (*Arctia plantaginis*) to those of their full sibs having their distinctive orange‐black warning coloration. The results showed >290 differentially expressed genes genome‐wide. Genes involved in the immune system, structural constituents of cuticular, and immunity were mostly downregulated in the albino caterpillars. Surprisingly, higher expression was observed in core melanin genes from albino caterpillars, suggesting that melanin synthesis may be disrupted in terminal ends of the pathway during its final conversion. Taken together, these results suggest that caterpillar albinism may not be due to a depletion of melanin precursor genes. In contrast, the albino condition may result from the combination of faulty melanin conversion late in its synthesis and structural deficiencies in the cuticular preventing its deposition. The results are discussed in the context of how albinism may impact individuals of aposematic species in the wild.

## Graphical abstract

Albinism in warningly colored caterpillars.

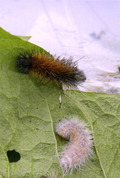

## 1 INTRODUCTION

Coloration is perhaps one of the most prominent adaptations for survival and reproduction of many taxa. It serves a variety of functions such as concealment for protection or ambushing, intra‐ or interspecific communication for sexual signaling or advertisement, and regulation of physiological processes such as body temperature (Cott, 1940; Endler & Mappes, 2017). Coloration can be produced by physical (i.e., structural) and chemical (i.e., pigments) means, and it can change with ontogeny and/or seasonality. Research on coloration goes beyond the evolutionary/ecological fields and has influenced breakthroughs in the design of new materials and technologies such as cosmetics and optical sensors (Caro et al., 2017).

Coloration is of particular importance for aposematic species, which rely on their coloring and patterning to act as a warning signal advertising unpalatability to predators (Rowe & Guilford, 2000). Such antipredator adaptation is widespread across plants and animals (Komarek, 1998; Mappes et al., 2005). Most research has focused on the evolution of warning coloration by natural selection where its effectiveness is relative to its intra‐ or interspecific frequency, and in the context of the predator community (Mappes et al., 1999; Merilaita & Kaitala, 2002; Mochida, 2011; Nokelainen et al., 2014; Papaj & Newsom, 2005; Pener & Simpson, 2009). At the genomic level, aposematic coloration and patterning have been extensively studied, particularly in insects (Van Belleghem et al., 2017; Brower, 2013; Dasmahapatra et al., 2012; Davey et al., 2016; Mallet, 2010; Zhan et al., 2014). However, almost no genomic information is available for color mutants in wild populations. This is mainly due to the difficulty of obtaining samples given their very low expected frequencies.

Color mutants provide a rare but excellent opportunity to confirm suggested pigmentation mechanisms, and to gain insight on alternative or complementary mechanisms involved in warning coloration and patterning. Mutations that have a major effect, such as a complete absence of pigmentation (i.e., albinism), can provide valuable information. Albinism and leucism (i.e., partial absence of pigmentation) are best studied in humans and in other mammals (Crawford et al., 2017; Martinez‐Garcia & Montoliu, 2013; Rothammer et al., 2017; de Vasconcelos et al., 2017). Nonetheless, such conditions can be found across taxa including fish (Clark, 2002; Nobile et al., 2016), reptiles (Krecsák, 2008; Mitchell & Church, 2002), and plants (Gettys & Wofford, 2007; Us‐Camas et al., 2017). In insects, albino strains of the silkworm (*Bombyx mori*) and the locusts pests *Locusta migratoria* and *Schistocerca gregaria* are commonly reared (Fujii et al., 2013; Sugahara et al., 2017). However, knowledge of albinism in wild insect populations is limited to cave‐dwelling species such as Carabid beetles (*Neotrechus* sp.) and the planthopper (*Oliarus polyphemus*) (Bilandžija et al., 2012). No information is available for aposematic insects.

The wood tiger moth (*Arctia plantaginis*) is an aposematic species widely distributed throughout the Holarctic. Adults are sexually dimorphic. Female hindwing coloration varies continuously from orange‐red, whereas male hindwing coloration varies within populations from yellow‐red in the Caucasus, yellow‐white in Europe and Siberia, and black‐white in North America and Northern Asia (Hegna et al., 2015). Caterpillars, however, are dichromatic displaying an orange patch against an otherwise black body, which functions as a warning signal against avian predators (Lindstedt et al., 2008). The patch is made of clusters of chitin hairs pigmented with eumelanin and diet‐derived flavonoids that give it its orange coloration (Lindstedt et al., 2010). The black hairs on the other hand contain only eumelanin (Lindstedt et al., 2016), a type of melanin that produces black pigmentation.

**FIGURE 1:**
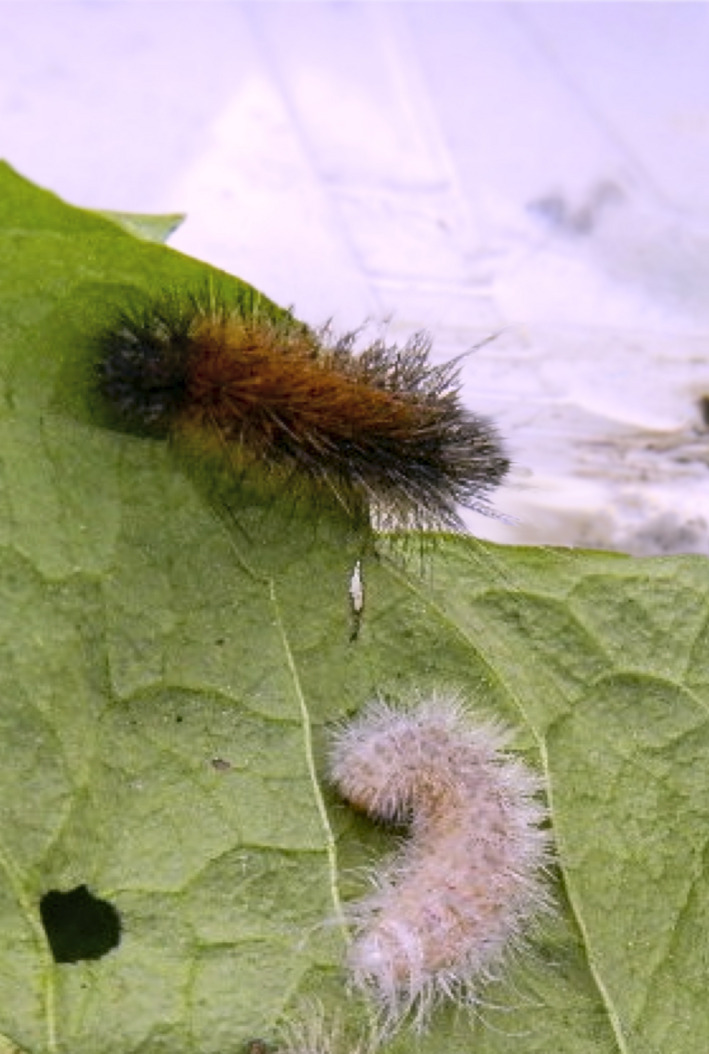
Albino and warningly colored wood tiger moth (*Arctia plantaginis*) caterpillars

Here, I compare the transcriptomes of albino mutant caterpillars of the wood tiger moth to those of their colored full sibs having their typical orange‐black warning coloration (Figure 1). I characterize genome‐wide gene expression differences and compare expression profiles of candidate genes from the melanin biosynthesis pathway. I further provide a comprehensive annotation of the differentially expressed genes. Finally, I discuss how albinism may impact individuals of aposematic species in the wild.

## 2 MATERIAL AND METHODS

Wood tiger moths have been reared at the University of Jyväskylä, Finland, since 2009. The stock is dynamic (i.e., not permanent selection lines) being supplemented every year with wild‐caught adults from which an average of 15,000 caterpillars are produced every year. Albino mutant caterpillars are rare, with only 8 cases documented showing completely white skin and hairs (Figure 1). Albino caterpillars usually die within a few days or, in some cases, regain their warning coloration in the next molt, suggesting that this condition can be lethal or transient.

### 2.1 Sample collection and RNA sequencing

In this opportunistic study, one albino caterpillar was collected in 2011, another in 2014, and a third one in 2018. All caterpillars were F1 from wild‐caught parents from central Finland. In each case, one of their normal colored full sibs was collected as well for comparative purposes. All caterpillars were in the fourth or fifth instar. They were fed with wild dandelion (*Taraxacum* spp.) and reared at the University of Jyväskylä, under natural light conditions with an average temperature of 22°C during the day and 15–20°C at night. For RNA extraction, whole caterpillars were submerged in RNAlater stabilizing solution (Qiagen, Valencia, USA) and kept at −20°C until extraction. Total RNA was extracted using RNeasy Mini Kit (Qiagen) according to the manufacturer's instructions with additional Tri Reagent (MRC, Inc.) and DNase (Qiagen, Valencia, USA) treatments. The quality and quantity of total RNA were inspected in a BioAnalyzer 2100 using RNA 6000 Nano Kit (Agilent). Subsequently, mRNA was isolated by means of two isolation cycles using Dynabeads mRNA Purification Kit (Ambion®) and quantified using RNA 6000 Pico Kit in a BioAnalyzer 2100 (Agilent). Pair‐end (2 × 100 pb) cDNA libraries were constructed for each caterpillar (i.e., 3 albino and 3 colored) according to Illumina's TruSeq Stranded HT protocol. The libraries were individually indexed and sequenced in a HiScanSQ sequencer in high‐throughput mode.

### 2.2 Reads processing and mapping

The quality of the raw reads from the libraries was first inspected with FastQC (http://www.bioinformatics.babraham.ac.uk/pro‐jects/fastqc/) and summarized using MultiQC v0.8 (Ewels et al., 2016). Based on this initial quality check, the FASTX toolkit (http://hannonlab.cshl.edu/fastx_toolkit/) was used to remove low‐quality bases and sequencing artifacts. Bases with a Phred quality score of less than 25 were filtered out, and reads shorter than 85 bases after trimming were removed. The remaining high‐quality reads were then sorted and synchronized using custom scripts.

### 2.3 Gene expression and annotation

To investigate gene expression differences between the two conditions, the high‐quality reads from the six samples were first mapped to the wood tiger moth's reference transcriptome (Galarza et al., 2017) using Bowtie 2 v. 2.2.5 (Langmead & Salzberg, 2012). The number of mapped reads for each sample was counted using SAMtools v.1.3.1 (Li, 2009) and merged into a count matrix. The clustering of the individual samples was inspected through principal component analysis (PCA), and multidimensional scaling (MDS) using the R packages factoextra v1.0.7 (Kassambara & Mundt, 2020) and edgeR v.3.30.3 (Robinson et al., 2010), respectively. The R package edgeR v.3.30.3 (Robinson et al., 2010) was used to test for differential expression at the gene level under a paired with blocking design, setting a cutoff threshold of Log2FC ≥ 2 and *p*‐value ≤ .05, and a Benjamini and Hochberg correction for multiple testing (Benjamini & Hochberg, 1995). The general distribution of both Log2FC and corresponding *p‐*values was assessed through a volcano plot showing the gene expression profiles of albino samples relative to colored samples (Figure S1). Subsequently, a functional annotation of the differentially expressed genes was obtained by blasting (BLASTx) (Altschul et al., 1997) against National Center for Biotechnology Information (NCBI) nonredundant protein databases (nr) (last accessed 13–10–2020) and the Swiss‐Prot database (last updated 25–06–2020). All hits that showed <70% of amino acid identity, alignment length of <100 bp, and e‐value of ≥10^–5^ were excluded. Gene ontology terms (GO) and information of protein family were obtained using Blast2GO v.4.0 (Conesa et al., 2005).

### 2.4 Gene expression validation

To validate gene expression results from the RNA‐seq data, a subset of three differentially expressed transcripts involved in cuticular processes was evaluated through quantitative PCR (qPCR). Likewise, another three unannotated differentially expressed genes selected at random were also included in the qPCR analyses to confirm that no artifacts could be biasing gene expression patterns. Exon–intron boundaries were first identified by aligning the transcripts to wood tiger moth genomic data previously obtained through 454 pyrosequencing (Life Sciences) using Mummer v.3.23 (Kurtz et al., 2004). Bridging primers were then designed using Primer3 v. 4.0.0 (Untergasser et al., 2012). As a normalization controls (i.e., housekeeping genes), I selected one transcript (TRINITY_DN29483_c0_g2_i1) from the RNA‐seq data, which showed a uniform expression level within and between the two larval conditions, and a gene (GADPH) known to have stable expression in moth species in a variety of conditions (Lu et al., 2013). The software NormFinder v.5 (Andersen et al., 2004) was used to evaluate the normalized count matrix to find the transcript with the highest stability value and lowest expression variation within and between the two conditions. The annotation and primer sequences of the transcripts used for qPCR are presented in Table S1.

RNA for qPCR validation was extracted and purified as described above from the same caterpillars of both conditions. High‐quality RNA (200 ng) was converted into cDNA using iScript cDNA Synthesis Kit (Bio‐Rad). The specificity, dynamic range, and PCR efficiency of each primer pair were determined by testing against a 6‐step twofold dilution series of cDNA. All genes were amplified by triplicate (i.e., as technical replicates) in the three caterpillars from each condition (i.e., as biological replicates). All qPCRs (20 µl final volume) were run on a CFX96 (Bio‐Rad™) thermocycler using 300 nM of each primer, 10 µl iQ SYBR^®^ Green Supermix (Bio‐Rad™), and 4 µl of cDNA diluted fourfold. PCR conditions used throughout were 95°C for 3 min followed by 40 cycles of 95°C for 10 s, 60°C for 15 s, and 72°C for 10 s. Melt curves were run after amplification to check for specificity from 55°C to 95°C with fluorescence readings taken in 0.5°C increments. Amplification efficiency of each gene was evaluated by plotting the standard curve Cq values against the log of the dilution factor for each point on the curve. The relative change in gene expression between the treatments was examined using the delta Ct method (Schmittgen & Livak, 2008).

### 2.5 Candidate melanin biosynthesis genes

Given that eumelanin is the dark pigment in larvae (Lindstedt et al., 2010), here I evaluate differences in expression profiles of candidate genes from the melanin biosynthesis pathway. The genes investigated were tyrosine hydroxylase (*TH*)*, yellow*, *laccase2*, dihydroxyphenylalanine‐DOPA decarboxylase (*Ddc*), arylalkylamine N‐acetyltransferase (*aaNAT*), *tan*, and *ebony*. These genes or the enzymes they encode have been shown to impact black pigmentation and pattern formation in a number of insect taxa (Fujii et al., 2013; Liu et al., 2016; van't Hof & Saccheri, 2010). In addition, I evaluate the expression of 6‐pyruvoyl‐tetrahydropterin (*PTS*), a gene outside the melanin pathway that encodes tetrahydrobiopterin (BH4), a cofactor that impacts the hydroxylation of tyrosine, the precursor of melanin synthesis. It has been shown that mutations in *PTS* can promote albinism in the silkworm (*Bombyx mori*) (Fujii et al., 2013).

Lepidoptera mRNA sequences from each candidate gene were obtained from NCBI and searched for orthology in the wood tiger moth reference transcriptome (Galarza et al., 2017) through their protein translation to all six possible frames using tBLASTx, and then blasted (BLASTx) back to NCBI to confirm orthology. Finally, the expression of the ortholog transcripts in each caterpillar condition was evaluated. For each transcript, the number of mapped reads (Mr) was divided by the total number of reads (Tr), multiplied by transcript length (Tl), and scaled by a factor of a million (i.e., $\left(\text{Mr/Tr}\right)*10^{9}$). This procedure returns normalized counts as transcripts per every million reads sequenced (TPM), in which the sum of all TPMs in each sample is the same, thus allowing a direct comparison of normalized expression values across samples and conditions. A Wilcoxon test was implemented in R v3.3.3 (R Core Team, 2017) setting the *paired* argument to TRUE and a significance level of *α* = 0.05 for the null hypothesis of no differences between mean TPM values of the two caterpillar conditions. The candidate gene transcript sequences and the orthologs’ accession numbers are given in the Table S2.

## 3 RESULTS

### 3.1 Gene expression, validation, and annotation

**FIGURE 2:**
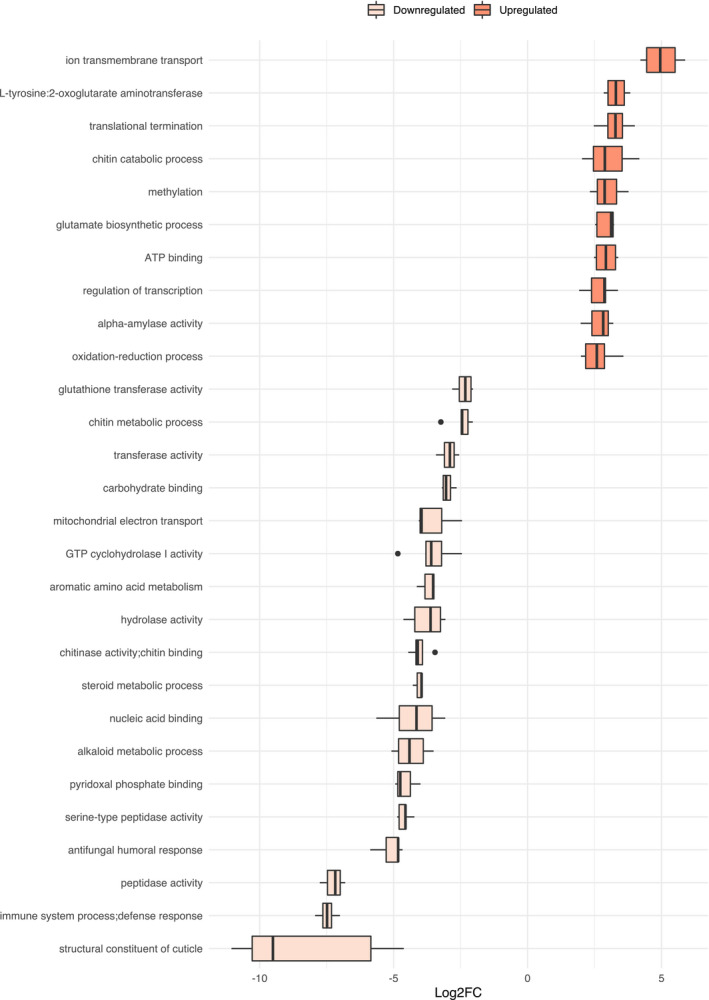
Differentially expressed genes (LogFC ≥ 2, *p* ≤ .05**)** grouped by functional category (GO terms) in albino and colored wood tiger moth (*Arctia plantaginis*) caterpillars. Shown are the Log2FC values of genes being expressed. Positive and negative Log2FC values indicate up‐ and downregulated genes, respectively, in albino caterpillars relative to warningly colored ones

A mean of 12.2 M (min 4.7, max 19.7) and 18.7 M (min 8.3, max 29.1) high‐quality (Phred ≥ 25, length > 85bp) paired reads per sample was obtained after trimming and filtering for the colored and albino samples, respectively. All read data can be found at National Center for Biotechnology Information (NCBI) under Project Number PRJNA449279 with Accession GGLW00000000. Both unsupervised clustering methods, MDS and PCA, separated the samples according to their phenotype. The warningly colored samples showed a tighter grouping, whereas the albino samples appeared more dispersed from each other, but separate from the warningly colored ones (Figure S1). A total of 302 genes were differentially expressed (LogFC ≥ 2, *p* ≤ .05) between the two conditions (Figure S1), of which 222 had functional annotation in public databases (Table S3). Grouping of these genes by their functional annotation (GO terms) showed a greater variety of biological processes downregulated in albino caterpillars (Figure 2). Genes involved in cuticular structure, chitin metabolism, and immune‐related processes such as defense and antifungal responses were downregulated in albino caterpillars, as indicated by their negative Log2FC relative to warningly colored caterpillars.

The qPCR validation of the candidate genes was in good agreement with the RNA‐seq data. All genes examined showed the same pattern of up‐ or downregulation in both data sets. Moreover, a significant correlation was observed (*r* = 0.94, *p* = .0046) between ΔCq form qPCR and the TPM values from RNA‐seq (Figure S2).

### 3.2 Candidate melanin biosynthesis genes

**FIGURE 3:**
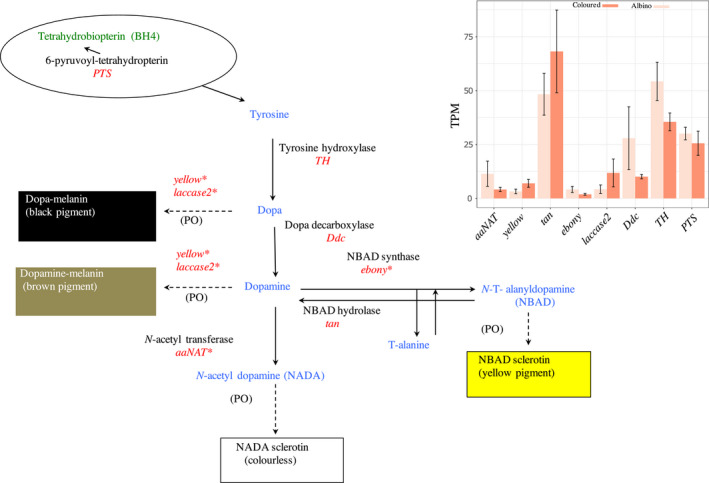
Insect melanin biosynthesis pathway. Green shows the cofactor outside the melanin pathway impacting the hydroxylation of tyrosine, the precursor of melanin biosynthesis. The precursor and pathway intermediates are shown in blue, enzymes in black, and the genes that encode them in red. Biochemical phenol oxidase reactions catalyzing pigment conversion are shown in dotted lines, and the final pigments produced are shown in boxes. Asterisk indicates the genes putatively involved in caterpillar albinism. Redrawn from Shamim et al. (2014), Futahashi et al. (2010), Ferguson, Green, et al. (2011). Inset bar chart shows the expression (TPM) of melanin genes in albino and warningly colored wood tiger moth (*Arctia plantaginis*) caterpillars

Contrasting results were observed in the expression of candidate melanin genes. Four genes (*aaNAT, ebony*, *Ddc, and TH*) showed significantly higher (*p* < .05) expression in the albino condition, whereas three others (*yellow*, *laccase2, and tan*) were more expressed in the colored condition. *PTS* on the other hand showed nonsignificant differences (*p* = .089) in its expression levels between conditions (Figure 3, Figure S3).

## 4 DISCUSSION

In this study, I report the transcriptome characterization and comparison between albino wood tiger moth caterpillars and their warningly colored full sibs. The results showed substantial differences genome‐wide. Processes such as immune response and cuticular formation were found significantly reduced in albino caterpillars. Albinism also appears to be associated with depressed immunity. Moreover, core melanin genes (*TH*, *Ddc*) showed a higher expression in albino caterpillars. However, a downregulation of genes (*yellow*, *laccase2*) involved in final melanin conversion was observed. Taken together, these results suggest that caterpillar albinism may not be due to a depletion of melanin precursor genes as it could be expected. In contrast, the albino condition may result from the combination of faulty melanin conversion late in its synthesis and structural deficiencies in the cuticular preventing its deposition.

### 4.1 The insect melanin biosynthesis pathway

Previous research has indicated that insect pigmentation results from both regulatory and structural genes, with many of the structural genes encoding enzymes that are involved in the biochemical pathways that generate pigments. The genes and their biochemical transformations within the melanin synthesis pathway in Lepidoptera are highly conserved, and their spatiotemporal expression determines the location and abundance of pigmentation (Ferguson et al., 2011; Futahashi et al., 2010; Shamim et al., 2014; Yu et al., 2011). As illustrated in Figure 3, insect melanization starts from the hydroxylation of tyrosine to 3,4‐dihydroxyphenylalanine (DOPA) encoded by the gene *TH*. Phenol oxidase (PO) catalyzes the conversion of DOPA to dopamine melanin encoded by *yellow* and *laccase2* genes, producing black melanin. In a parallel branch, DOPA can be transformed to dopamine through decarboxylation encoded by *Ddc* to produce dopamine melanin, a brown melanin. It can be expected that albino caterpillars suffer from a suppression or depletion of these genes given their lack of melanic pigmentation. However, *TH* and *Ddc* showed higher expression in the albino caterpillars (Figure 3). Moreover, outside the melanin pathway, *PTS,* the cofactor impacting the melanin precursor *tyrosine,* did not show differences between the two larval conditions (Figure 3). This indicates a nondepletion of master genes (*TH*, *Ddc*, *PTS*) in albino caterpillars, suggesting that melanin synthesis may be disrupted during the conversion of DOPA and dopamine to melanin by *yellow* and *laccase2* genes. The observed low expression of these genes in albino caterpillars further supports this notion (Figure 3). Functional studies are needed to investigate biochemical reactions such as oxidation–reduction regulating PO and their impact on the melanin‐promoting genes *lacasse2* and *yellow* family genes.

Downstream the melanin pathway, the production of dopamine melanin can be suppressed by converting dopamine to *N*‐b‐alanyldopamine (NBAD). In this branch, β‐alanine binds to dopamine by the activity of *ebony* forming NBAD, the precursor of yellow sclerotin, resulting in a yellow pigment. This can be reversed by the activity of *tan*, by which dopamine is converted back to dopamine melanin, thus promoting dark pigmentation. Finally, dopamine also can be converted to NADA sclerotin, which is colorless and encoded by *aaNAT*. Previous studies in Lepidoptera and other insects have found an upregulation of *ebony* and *aaNAT* in nonblack body regions, while *tan* is generally expressed in black regions (Ferguson et al., 2011; Futahashi et al., 2008; Liu et al., 2016; Osanai‐Futahashi et al., 2012; Zhan et al., 2010). This is congruent with the results obtained here where the melanin‐promoting genes *tan* and *yellow* showed a higher expression in the colored caterpillars, whereas the melanin‐suppressing genes *ebony* and *aaNAT* were more expressed in the albino caterpillars (Figure 3). Thus, in addition to a defective conversion of melanin from DOPA and dopamine upstream the pathway, melanin may be further suppressed downstream the pathway in albino caterpillars.

Sclerotization is an independent but parallel process to melanization used by insects for cuticular hardening and tanning. Freshly made cuticular can be pale and soft, turning harder and sometimes darker within a few hours after molting. Melanin is deposited into the cuticular via epidermal cells. The cuticular (i.e., exoskeleton) is composed of cross‐linked chitin fibers and matrix protein forming an effective barrier against harmful influences from the environment such as desiccation, microorganisms, and predators (Andersen, 2010). Chitin is a main component of the cuticular of insects, crustaceans, and arachnids providing rigidity and articulation. Its turnover is regulated by two main enzymes, chitin synthase for its synthesis and chitinase for its degradation (Merzendorfer & Zimoch, 2003). Although no systematic cuticular assessments could be conducted, the cuticulars of the albino wood tiger moths appeared softer and thinner than those of warningly colored ones, suggesting deficient sclerotization, which may prevent melanin deposition. This is also indicated by the downregulation of genes involved in chitin metabolic processes, structural constituents of cuticular, and the upregulation of chitin catabolic processes, which break down chitin (Figure 2, Figure S5). Electron microscopy from albino caterpillars of the silk moth (*Bombyx mori*) showed an abnormally loose cuticular structure compared with that of colored ones, resulting in an inability to chew because of insufficient hardening of their mandibles (Tsujita & Sakurai, 1971). Similar observations have been made in experimental unpigmented mutants (via RNA interference) of the red flour beetle (*Tribolium castaneum*) that showed a significant decrease in cuticular hardness (Gorman & Arakane, 2010). Thus, structural deficiencies in albino cuticulars may make them vulnerable to their surrounding environment and can complicate some fundamental activities such as feeding, all contributing to their low survival.

### 4.2 Albinism frequency

Albinism in humans has been extensively studied, and its frequency is well documented for distinct populations among different geographic regions (Montoliu et al., 2014). For wild species, however, little information is available about its prevalence. Albinism seems to be a common phenomenon in species living in lightless environments such as caves or subterranean habitats (Bilandžija et al., 2012, 2017; Oliveira & Aguiar, 2008; Protas et al., 2006). However, it can be observed also in aboveground species. For instance, albino chorus frogs (*Pseudacris triseriata*) have been reported in frequencies of 7% and 12% during two consecutive years from natural ponds (Corn, 1986). Likewise, scatter reports of albino Viperinae (*Vipera ammodytes*, *V. aspis*, *V. seoanei*, and *V. berus*) in Europe have been collected ranging from 1 to 16 observations depending on the species with an increase in frequency toward Nordic populations (Krecsák, 2008). In Lepidoptera, cases of partial albinism have been observed in alpine butterflies, *Erebia cpiphron silesiana* and *E*. *sudeiica sudetica,* with a frequencies ranging from 0.03%–1.4% in *E*. *cpiphron* to 0.7%–3.9% in *E. sudeiica* (Kuras et al., 2001). In the case of the wood tiger moth, no albinos have been observed in the wild. However, albino caterpillars from the rearing program are F1 from wild‐caught parents, from which, one in every 5,000 caterpillars displays the albino condition. This may serve as a lower‐end proxy of albino frequencies in the wild, but it needs to be better established by field studies.

### 4.3 Albinism and predation

It can be envisaged that albinism would have mostly adverse effects on antipredator strategies such as aposematism. However, this has not been examined in depth. In insects, there are only few predation experiments reported. Using four different wild‐caught bird species (*Parus major*, *Parus caeruleus*, *Erithacus rubecula,* and *Sylvia atricapilla*) as predators, Exnerová et al. (2006) found that white‐black mutants of the aposematic firebug (*Pyrrhocoris apterus*) were more attacked as the red‐black wild‐type and equally attacked as nonaposematic gray‐black controls. On the other hand, in a later experiment, naïve, hand‐reared *P*. *major* did not show any avoidance and attacked firebugs equally irrespective of color (Svadová et al., 2009). Data from a field study in two alpine butterflies (*E*. *cpiphron and E. sudeiica*) showed that predation marks and malformations were positively associated with albinism in both species (Kuras et al., 2001). It is worth noting that these studies examined partial albinos having some degree of dark pigmentation. The effect of predation on wholly albinos like those reported here remains to be examined. As suggested by the previous studies (Exnerová et al., 2006; Svadová et al., 2009), an effective aposematic strategy relies in great part on predator learning and generalization. It is unclear whether albino caterpillars would benefit from the effect of novelty or whether they are beyond the limits of predators’ scope of generalization. The effect of albinism regarding non‐visual predators such as ants and other invertebrates is also unknown. More studies are needed to investigate this. However, this is a difficult task due to the rarity of albino individuals, particularly in wild populations. Nonetheless, given the very low frequencies of albinism it can be inferred that the albino condition has severe adverse effects on the survival/fitness of aposematic species.

### 4.4 The importance of dark pigmentation

Dark pigmentation plays a fundamental role in aposematic insects as both dark and bright colors in a contrasting pattern are necessary to display an effective warning signal. In wood tiger moth caterpillars, the orange patch contrasts with the dark body conforming the warning signal, which is more effective against visual predators when the orange patch is large (Lindstedt et al., 2009). The orange pigment results from diet‐derived flavonoids (Lindstedt et al., 2010). Flavonoids are one of the major groups of plant secondary metabolites including over 9,000 compounds, and their biological activity depends, to a large extent, on their structural diversity (Alzand, 2012). Their role in the moth's physiology is currently unknown. Metabolomic studies combined with transcriptome analyses are needed to gain mechanistic insight on the processes they might impact.

Melanin on the other hand (i.e., the dark pigmentation) is central for insect immunity (Tsakas & Marmaras, 2010). The innate immune system of insects consists of physical barriers such as the integument and the peritrophic membrane, as well as humoral and cellular responses (Rosales, 2017). When infected, hemocytes such as plasmatocytes and granulocytes transported by the hemolymph are activated leading to phagocytosis, nodule formation, and encapsulation (Jiang et al., 2010). Invading microorganisms are recognized by pattern recognition protein receptors (PRPs) that bind conserved domains located on the lipids and carbohydrates synthetized by the invading microorganisms (Kanost & Trenczek, 1997). Serine proteinases then stimulate the activation of the Toll and immune deficiency (IMD) pathways for the expression of antimicrobial peptides (AMPs), mainly synthetized by the fat body and secreted into the hemolymph (Casanova‐Torres & Goodrich‐Blair, 2013; Jiang et al., 2010; Rosales, 2017).

Although melanic dark pigmentation generally correlates with insects’ immunity, these two processes appear to affect albino caterpillars independently. In looking at the totality of expressed genes, melanin genes showed a pattern of upregulation, while genes in the insect canonical immune system were found mostly downregulated (Figs S4, S5, Table S4). However, these results should be taken with caution given the large variation in Log2FC values. A cutoff of Log2FC ≥ 2 and *p*‐value ≤ .05, with a Benjamini and Hochberg correction for multiple testing (Benjamini & Hochberg, 1995), is shown in Figure 2. Nonetheless, Figures S4 and S5 and Table S4 suggest that multiple genes of small effect could potentially have an impact on the melanization and immunity of albino caterpillars.

Experiments with high‐ and low‐melanin wood tiger moth caterpillars have shown a better resistance to oral bacterial infections in high‐melanin caterpillars (Zhang et al., 2012). Likewise, more melanized caterpillars showed a faster encapsulation response to artificial implants than less melanized ones (Nokelainen et al., 2013). By analogy, the complete absence of melanization in albino caterpillars hints to a suppressed immunity. This notion is supported by the gene expression results showing a downregulation of genes involved in immune system, defense response, and antifungal responses in the albino caterpillars (Figure 2). However, it has recently been shown that wound‐healing melanization can still occur in most albino cave‐adapted adapted species, including insects (Bilandžija et al., 2017). Whether this is the case in albino wood tiger moth is unknown. Future studies should investigate possible melanization responses to wounding or macroparasites in albinos from aboveground species.

Melanic dark pigmentation also protects from deleterious solar radiation and at the same time helps in thermoregulation (Ellers & Boggs, 2004; Stoehr & Goux, 2008). It has been shown that darker wood tiger moth caterpillars are more efficient in thermoregulation than caterpillars with larger orange patches (Lindstedt et al., 2009). More recently, it was found that darker caterpillars absorb more heat, keep higher body temperature, and actively avoid overheating by seeking shade sooner than less melanized caterpillars (Nielsen & Mappes, 2020). Such behavioral variation relative to the amount of dark pigmentation could be expected to be exaggerated in albino caterpillars. Accordingly, albinos may need partially sheltered thermoregulation due to the lack of protective dark pigmentation. This could result in longer heat‐up periods, which in turn may reduce foraging time and also increase their exposure to nonvisual predators. If true, albinos may face different predation pressures than their warningly colored counterparts, for which, an aposematic strategy may be irrelevant.

## 5 CONCLUSION

It is well recognized that neurosensorial, biochemical, metabolic, and physiological anomalies are associated with the albinism condition in mammals (Creel, 1980; Oetting & King, 1999). Albino insects seem to suffer from similar pathological conditions. The albino caterpillars studied here showed a substantial differentiation in biological processes and metabolic functions compared with their warningly colored full sibs. Some symptoms can have severe deleterious effects in their physiology (i.e., suppressed immunity) and in their structural properties (i.e., defective cuticular sclerotization). The occurrence of albinism in wildwood tiger moth populations and its effect on the aposematic strategy of the species are unknown. However, its frequency can be expected to be very low, and presumably, albinos experience different selection pressures due to behavioral differences induced by their peculiar physiology. More field surveys and experimental studies are needed to explore these notions.

## AUTHOR CONTRIBUTION

**Juan A Galarza:** Conceptualization (lead); Formal analysis (lead).

## Supporting information


FigS1

FigS2

FigS3

FigS4

FigS5

TableS1

Supinfo

## Data Availability

All sequence data can be found at National Center for Biotechnology Information (NCBI) under Project Number PRJNA449279 with Accession GGLW00000000.
