# Supplementary figures and images for: Comparative transcriptomics of albino and warningly‐coloured caterpillars
Source: Ecol Evol. 2021 May 2;11(12):7507–17. doi: 10.1002/ece3.7581 (PMC8216890; doi:10.1002/ece3.7581)

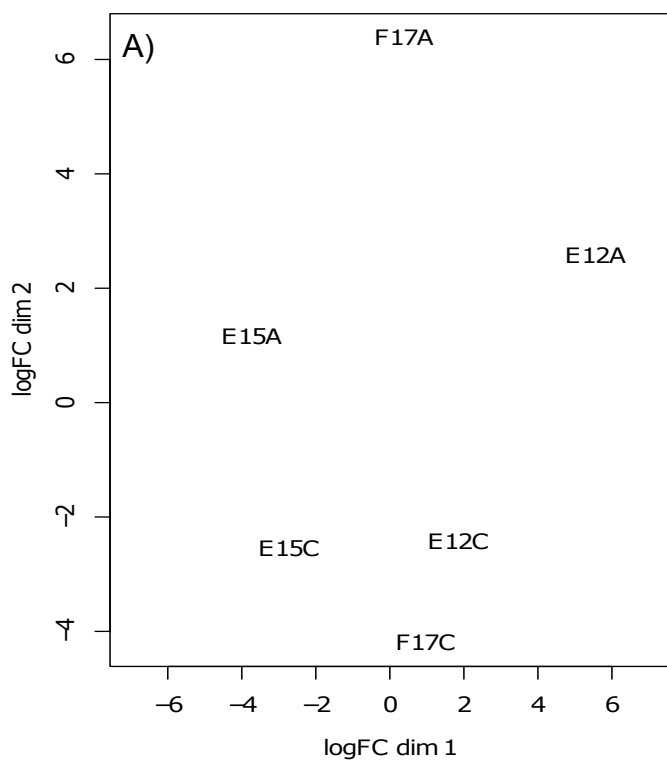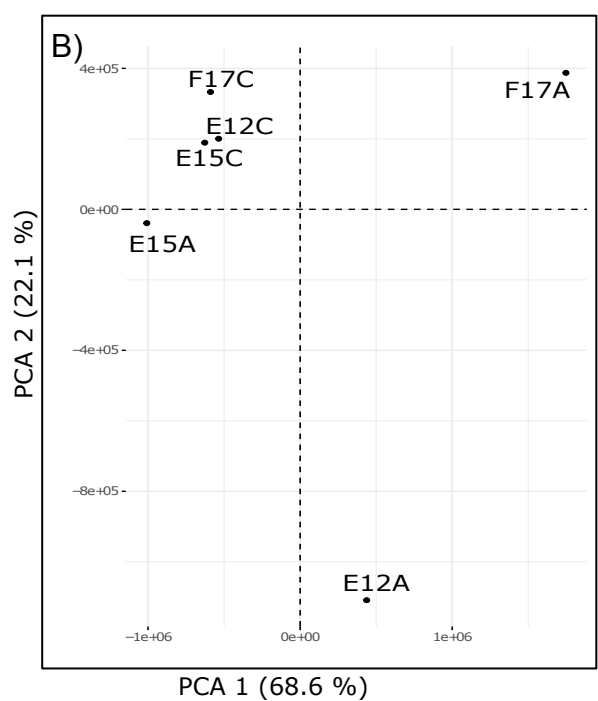

C)      ● NS    ● p-value    ● p-value and log<sub>2</sub> FC

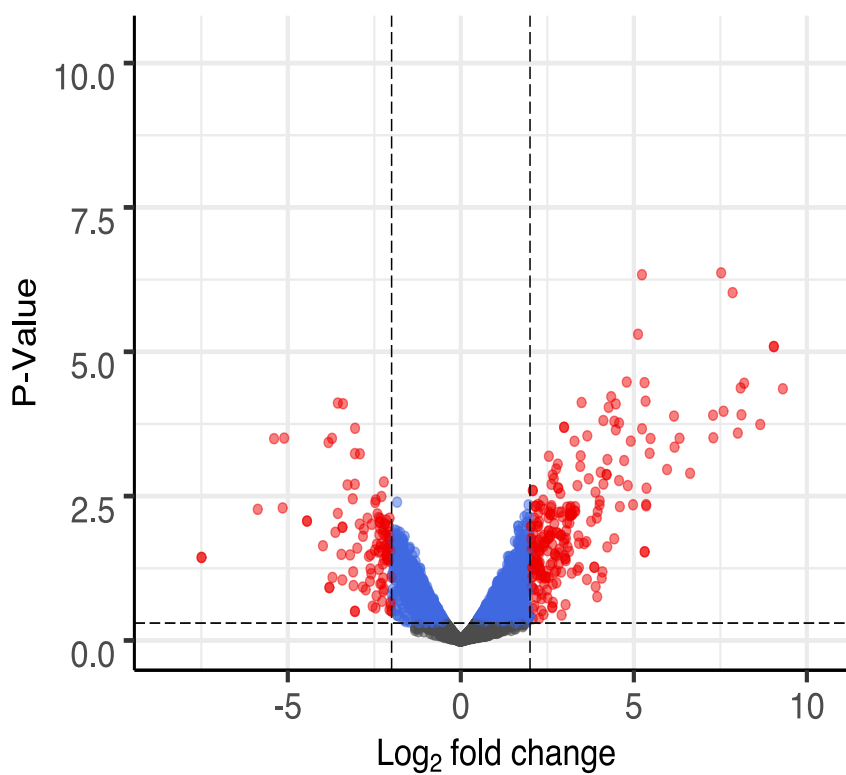

Supplement: Supplementary file 1 — FigS1 [file ECE3-11-7507-s003.pdf]

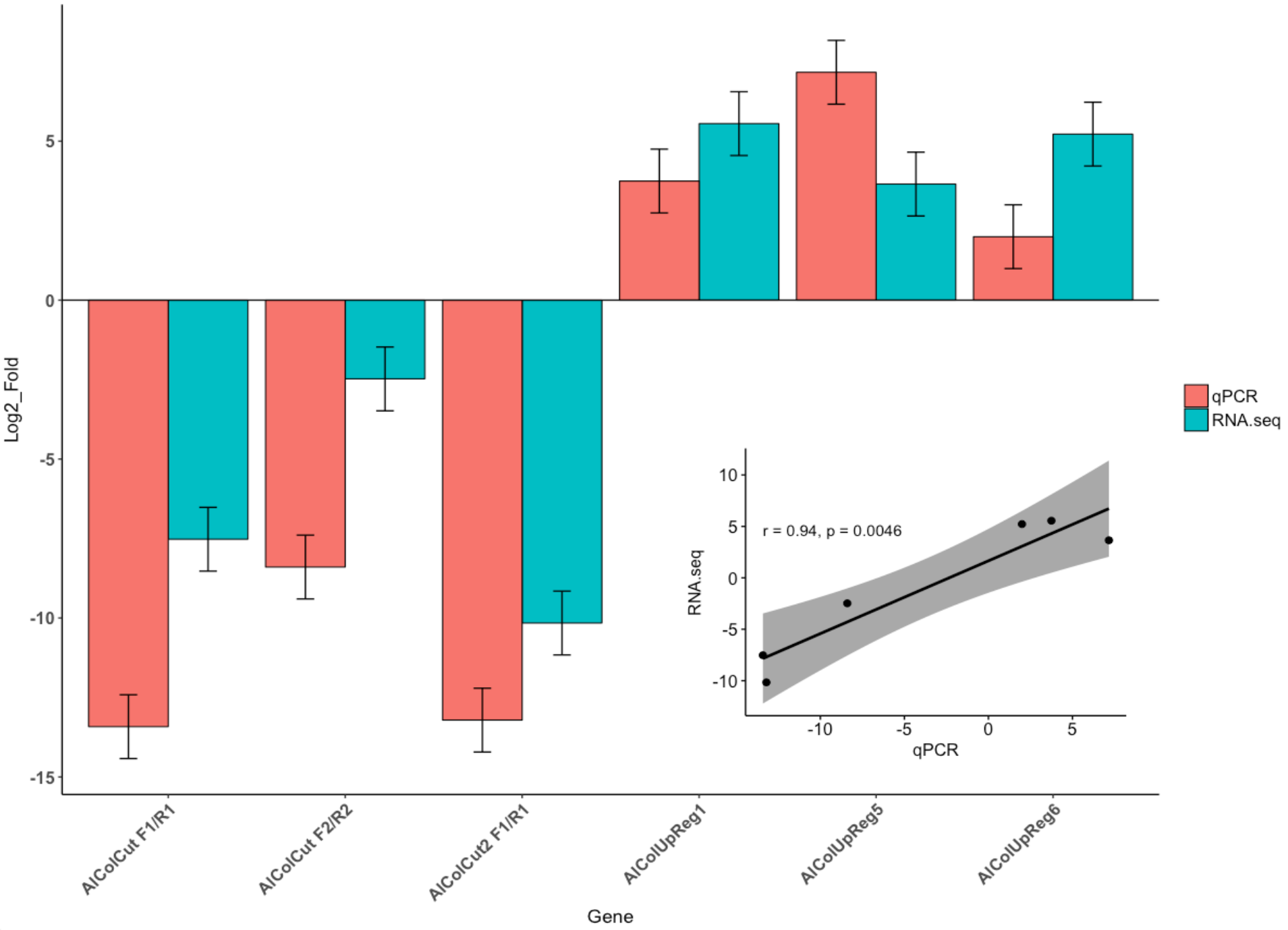

Supplement: Supplementary file 2 — FigS2 [file ECE3-11-7507-s005.pdf]

*aaNAT* \*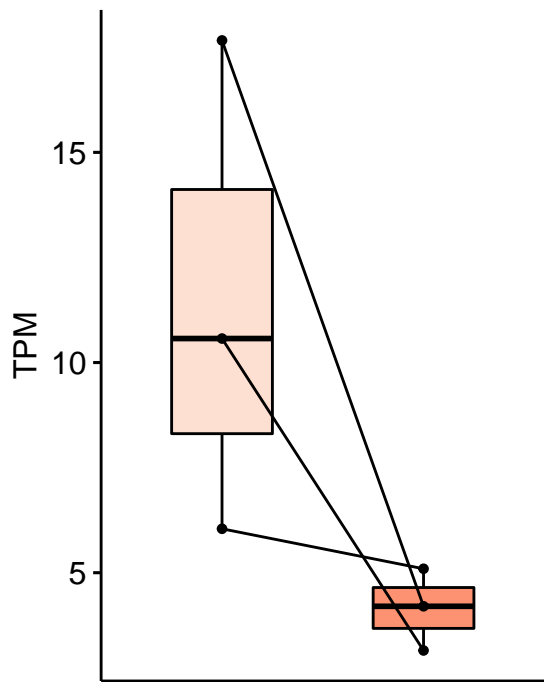*yellow* \*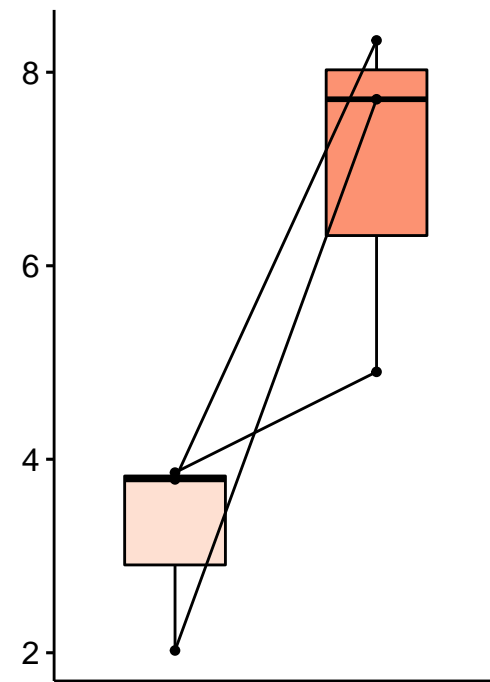*tan* \*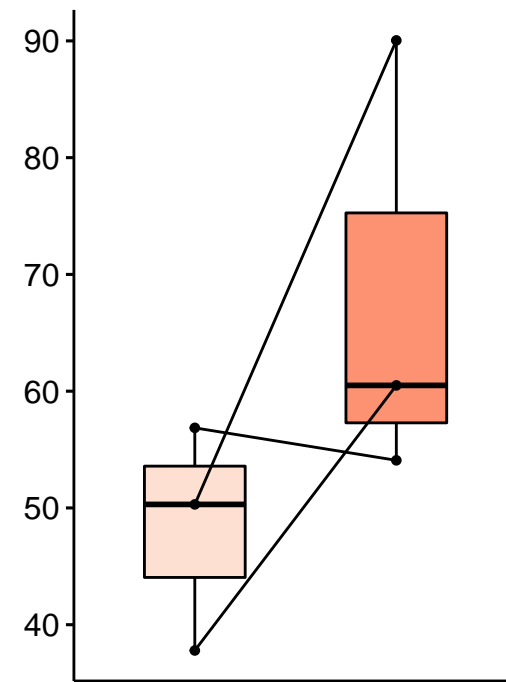*ebony* \*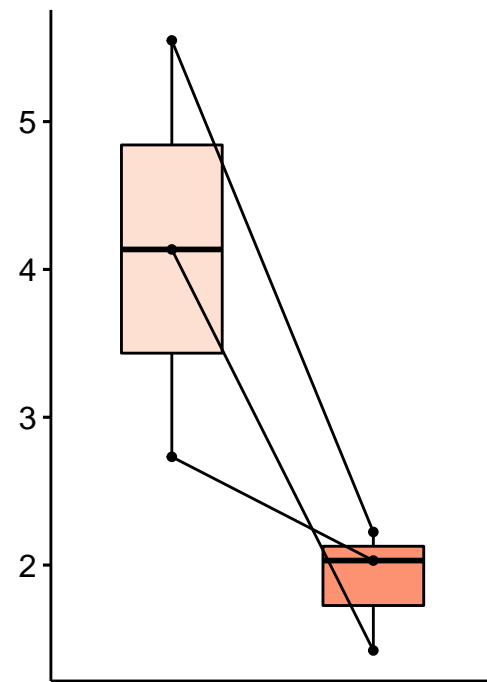*laccase2* \*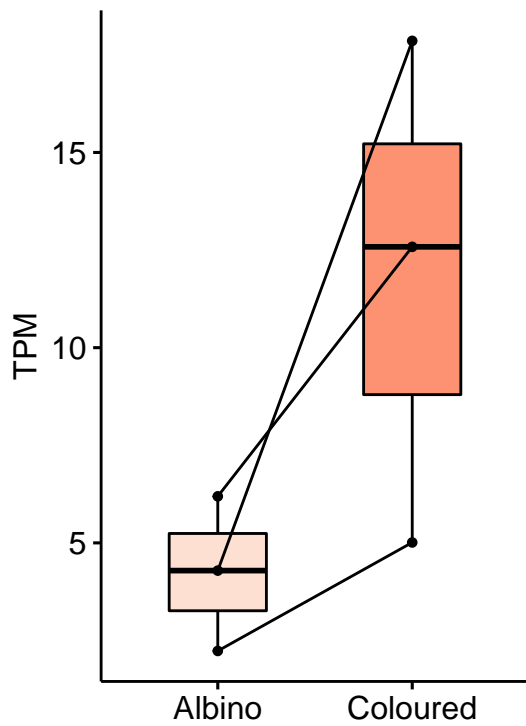*Ddc* \*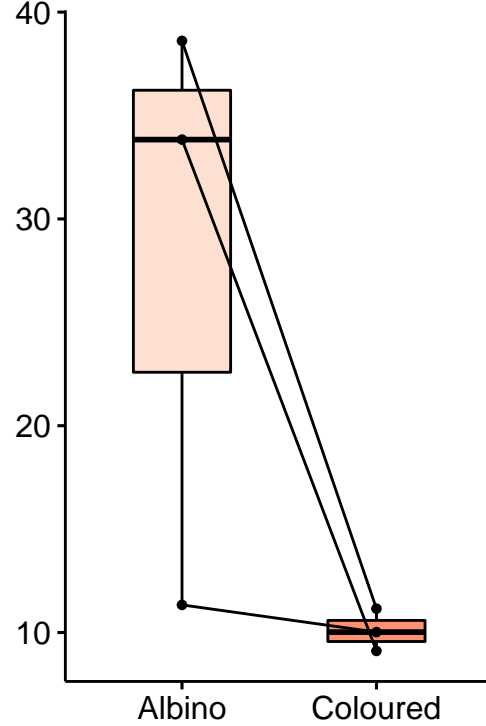*Th* \*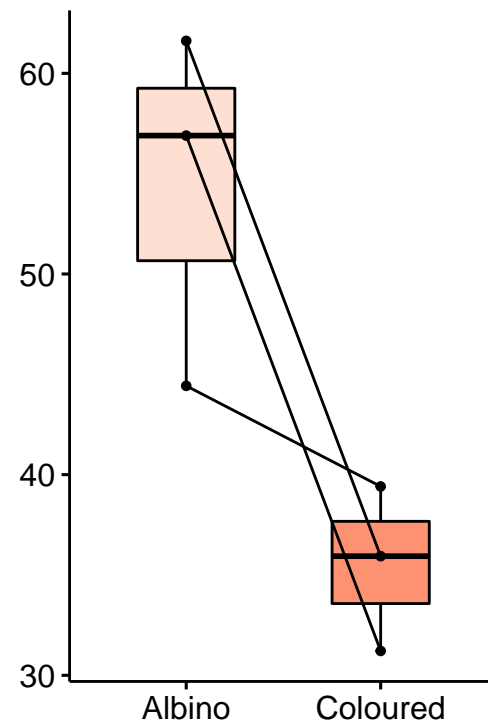*PTS* <sup>NS</sup>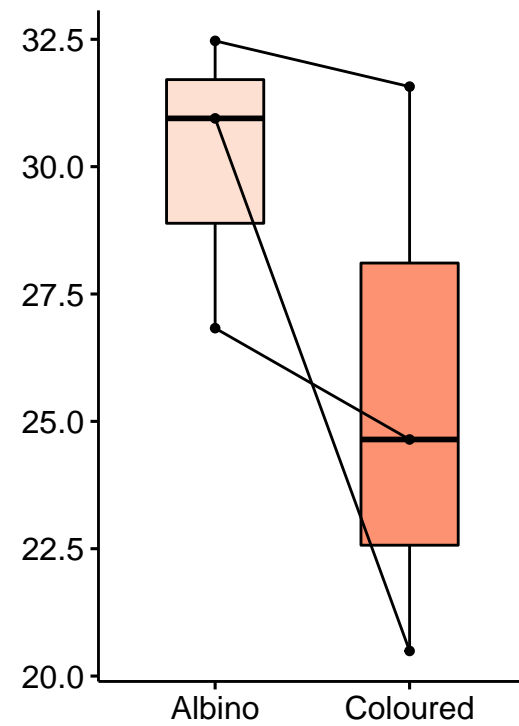

Supplement: Supplementary file 3 — FigS3 [file ECE3-11-7507-s002.pdf]

Log2FC

5 -

0

-5

Transcripts

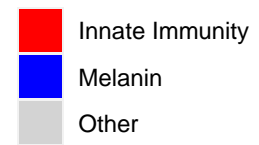

Supplement: Supplementary file 4 — FigS4 [file ECE3-11-7507-s007.pdf]

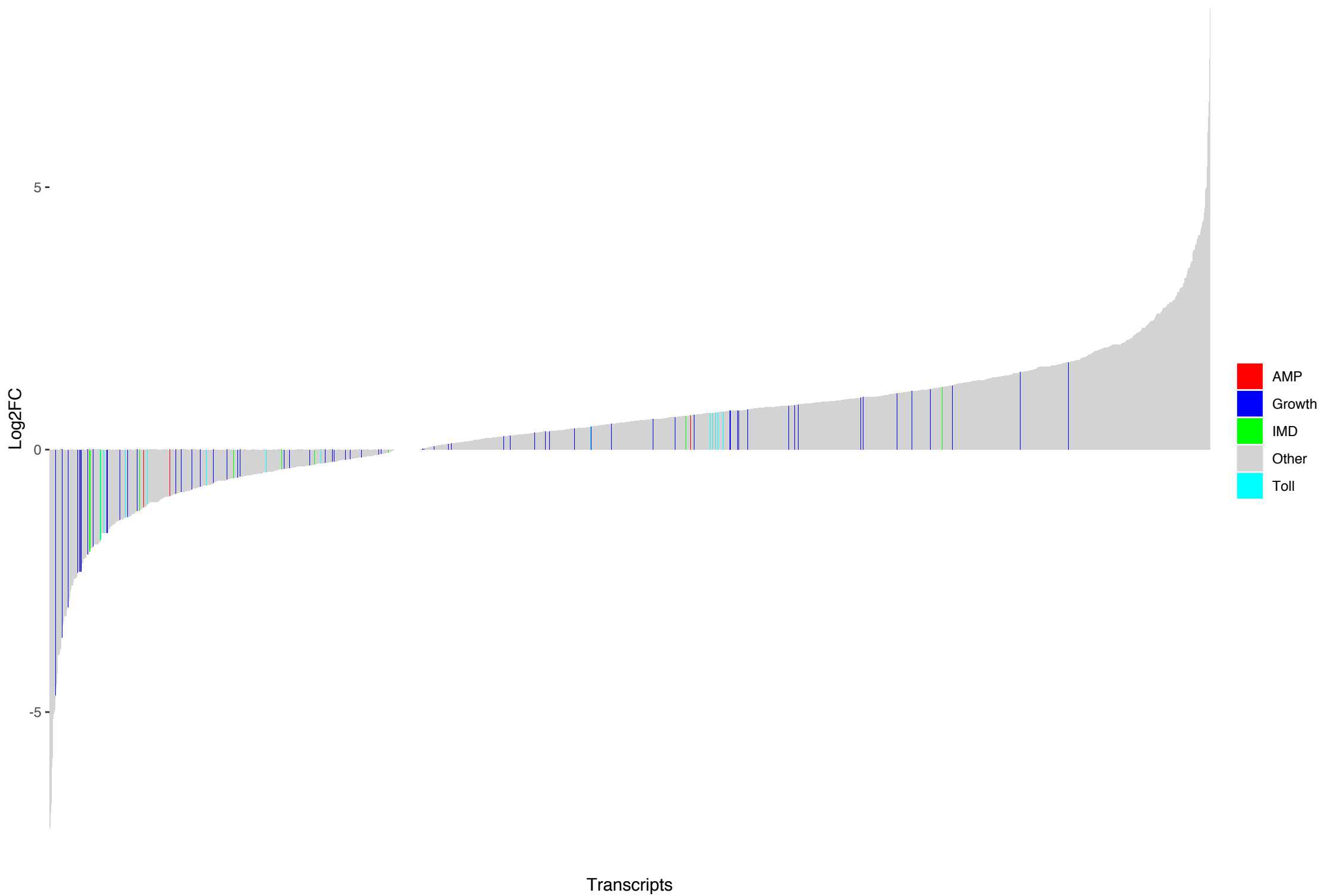

Supplement: Supplementary file 5 — FigS5 [file ECE3-11-7507-s001.pdf]
